# Naturalistic examination of the anxiolytic effects of medical cannabis and associated gender and age differences in a Canadian cohort

**DOI:** 10.1186/s42238-023-00192-x

**Published:** 2023-06-09

**Authors:** Meenu Minhas, Stephanie E. Lunn

**Affiliations:** 1 Aurora Cannabis Inc, Edmonton, AB Canada; 2 Aurora Cannabis Inc, 1590 Galbraith Rd, BC V9M 4A1 Comox, Canada

**Keywords:** Cannabis, Anxiety, Sex differences, THC, CBD, Real-world data

## Abstract

**Background:**

The aim of the current study was to examine patterns of medical cannabis use in those using it to treat anxiety and to investigate if the anxiolytic effects of cannabis were impacted by gender and/or age.

**Methods:**

Patient-reported data (*n* = 184 participants, 61% female, 34.7 ± 8.0 years) was collected through the Strainprint^®^ app. Tracked sessions were included if the method of administration was inhalation, treatment was for anxiety and the product used was dried flower. The final analyzed dataset encompassed three of the most commonly utilized dried flower products in anxiety sessions. Independent sample *t*-tests were used. The core analysis examined within subject changes overtime (pre-medication to post-medication) and interactions between time with two candidate moderators [gender (male, female) and age (18–29, 30–39, and 40 + years old)] by using analysis of variance (ANOVA). For significant main effects of interactions, post hoc tests were conducted using a Bonferroni correction. A secondary analysis examined differences in proportion of emotives endorsed as a function of gender or age using chi-square test of independence.

**Results:**

Cannabis consumption resulted in a significant decrease in anxiety scores among both males and females (average efficacy of 50%) and efficacy was similar across the three cultivars. However, gender differences in efficacy were identified in two of the cultivars. All age groups experienced significant reductions in their anxiety post cannabis consumption; however, the 40 + year old group had significantly less efficacy than the other groups. The overall optimal dosing for the entire cohort was 9–11 inhalations for males and 5–7 inhalations for females, with some variation in dosing across the different cultivars, genders and age groups.

**Conclusions:**

We found all three cultivars had significant anxiolytic effects and were well-tolerated. Some limitations of the study are the moderate sample size, self-reported diagnosis of anxiety, unknown comorbidities and experience with cannabis, whether other drugs or cannabis products were used, and restriction to solely inhaled administration. We suggest that the gender and age differences in optimal dosing could support both healthcare practitioners and patients initiate medical cannabis treatment for anxiety.

## Background

Anxiety disorders contribute globally to a total of 24.6 million years lived with disability (World Health Organization 2017). They are associated with a poor quality of life across various domains such as general health, physical health, body pain, mental health, as well as impairments in role and social functioning (Mendlowicz and Stein 2000; Barrera and Norton 2009; Hoffman, Dukes, and Wittchen 2008). Anxiety disorders encompass a number of conditions and symptoms that can range from mild to severe (“Mental Health - Anxiety Disorders - Canada.Ca” n.d.; Anxiety Canada n.d.; “What Are the Five Major Types of Anxiety Disorders? | HHS.Gov” n.d.). Although there are currently various anxiolytic pharmaceuticals available to patients, approximately a third will not respond to them (known as “treatment-refractory anxiety”) and only ~ 50% will fully recover (Garakani et al. 2020). Additionally, not all patient groups can tolerate the available pharmaceuticals. Benzodiazepines for instance, can be effective for some anxiety disorders but run the risk of tolerance, misuse, abuse and/or are riskier for older patients due to the risk of falling (Garakani et al. 2020). Thus, there is a need for new and effective treatments to aid these patients. With an increase in public awareness and scientific investigation, there is increasing interest in, and evidence for, the use of medical cannabis in patients who have not found success with existing anxiolytics.

Treatment of anxiety is among the top three endorsed motives for medical cannabis use (Wadsworth, Leos-Toro, and Hammond 2019; Walsh et al. 2013). In a real-world evidence study, 85% of patients with anxiety self-reported some improvement in their condition and had increased quality of life following six weeks of medical cannabis treatment (Cahill et al. 2021). Of those who rated cannabis as being helpful for their anxiety, 32% selected high cannabidiol (CBD) products, 28% selected high tetrahydrocannabinol (THC) products, and 8% selected balanced THC:CBD products (Cahill et al. 2021).

Numerous studies have shown CBD has therapeutic efficacy for reducing anxiety in those with generalized social anxiety disorder (Bergamaschi et al. 2011; Crippa et al. 2011), anxiety following an impromptu speech (Zuardi et al. 1993, 2017), and in clinical populations with anxiety (Shannon et al. 2019). For instance, CBD decreased anxiety scores within the first month in 79% of patients with anxiety and this improvement was sustained at the 3-month follow-up (Shannon et al. 2019). CBD-dominant treatments appear particularly beneficial in patients with moderate to severe anxiety (Rapin et al. 2021).

THC also has reported anxiolytic effects. Nabilone (a synthetic THC analog) has been shown to decrease anxiety in patients in two different studies (Fabre and McLendon 1981; Skrabek et al. 2008). Furthermore, a systematic review performed on 83 studies found that THC alone or in combination with CBD improved anxiety symptoms among individuals with comorbid medical conditions (Black et al. 2019).

While the above indicates cannabinoids show efficacy as anxiolytics, it is still not clear what specific THC:CBD ratio nor dose is most effective at treating anxiety and there continues to be gender differences reported for cannabinoid pharmacology (Tseng, Harding, and Craft 2004; Sholler et al. 2021; Spindle et al. 2020; Lunn et al. 2019) and anxiety pathophysiology (Toufexis et al. 2014; Kessler et al. 1994).

Females may be particularly vulnerable to anxiety disorders (Bandelow et al. 2014). Females are more likely than males to meet the criteria for anxiety disorders (McLean et al. 2011; Kessler et al. 1994, 1995) and less likely than males to respond to selective serotonin reuptake inhibitors for anxiety (Simon et al. 2006). This increased vulnerability is believed to be a result of female reproductive hormones and brain structure differences (Lebron-Milad and Milad 2012; Reimer et al. 2018).

Investigation into possible gender-related differences in the efficacy of medical cannabis have been limited, despite a significant proportion of females using medical cannabis (Government of Canada 2021) and doing so to treat anxiety (Moltke and Hindocha 2021; Cuttler, Mischley, and Sexton 2016). However, the available evidence does point to pharmacological differences amongst males and females in response to cannabis. Males are more likely to report memory improvement, to feel enthusiastic, experience an altered sense of time, experience munchies, less likely to have a desire to clean, and experience a loss of appetite(Cuttler, Mischley, and Sexton 2016) while females are more likely than males to report medical cannabis related adverse events (Aviram et al. 2021). When able to self-titrate, women smoked less cannabis and subsequently had lower THC plasma concentrations, while experiencing the same acute effects as males (Matheson et al. 2020). Nadulski et al. reported females had higher peak plasma concentrations (C_max_) and quicker time to peak plasma (T_max_) concentrations for THC, 11-hydroxy-THC (11-OH-THC) and CBD in comparison to males after orally ingesting a cannabis extract (Nadulski et al. 2005). Spindle et al. found female participants had higher plasma and oral fluid C_max_ for THC and its metabolites after eating THC brownies (Spindle et al. 2020) while Sholler et al. found females had a significantly greater C_max_ for 11-OH-THC than males post vaporized cannabis (Sholler et al. 2021).

Given that hepatic metabolism can be negatively impacted by aging (Klotz 2009; Tajir and Shimizu 2013), it is also possible age may play a role in how patients respond to medical cannabis. Taylor et al. found moderate-severe hepatic impairment significantly altered the pharmacokinetics of CBD and its metabolites’ and concluded CBD dosages should be modified for these patient groups (Taylor et al. 2019).

Thus, the goal of the current study was to examine changes in anxiety following medical cannabis use and how this may be impacted by gender or age. A secondary objective was to examine self-titration patterns over time and determine how dosing may be impacted by gender, age, and/or cultivar.

## Methods

### Study design

Archival data gathered between May 2017 and August 2021 was obtained from Strainprint^®^ and used to examine medical cannabis use for the management of anxiety. Strainprint^®^ is a free global mobile app, where medical cannabis consumers can track changes in the severity of their symptoms as a function of different doses, cultivars, and formats. To access medical cannabis in Canada, patients require a medical document from a healthcare practitioner (similar to a prescription) and must also be registered with a licensed producer of medical cannabis, who mails the product directly to patients.

During initial registration, individuals provided demographic information. Prior to medical cannabis use, Strainprint^®^ users tracked their sessions by selecting the symptom(s) they were experiencing at the time from a list of 279 conditions and 46 symptoms. The severity of each symptom was rated on a scale of 0 (least severe) to 10 (very severe). Consumers were then prompted to select the product they were going to use (e.g., producer or distributor of the cultivar) and their route of administration (smoke, oil, etc.) for that specific session. In the current study, a focus on inhalation as the method of administration was chosen given the acute onset of effects experienced from this format and its common use (Sexton et al. 2016). Only sessions that utilized Aurora Cannabis branded products were included as they are one of the largest suppliers of the Canadian medical cannabis market and focusing on one brand minimized any impact of differences in manufacturing practices between licensed producers. The final dataset included the three most commonly used Aurora Cannabis dried flower products for anxiety sessions: THC indica (17–19.1% THC: 0.1% CBD), CBD (0.3–2.5% THC: 8–10.3% CBD),and THC sativa (17.5–22.3% THC: 0–0.1% CBD). During their sessions, consumers indicated the dose (number of inhalations ranging from 1 to 10 +) self-administered during the session. Individuals were then prompted (via a push notification) to re-rate the severity of their symptom(s) 20 min after they consumed. The app engaged users through a loyalty reward system where users earned points for tracking sessions of cannabis consumption.

### Measures

#### Product efficacy

The efficacy score was calculated to determine the change in symptom severity. Efficacy was calculated by taking the average of two measures of change: Efficacy = ([(*x* − *y*)/*x*] + [(*x* − *y*) / 10])/2; where *x* represents severity prior to medicating and y represents severity post medicating.

#### Emotive responses

Participants were also asked to report self-perceived impacts following cannabis consumption through emotive-built assessments. Users chose from a list of positive (aroused, comfortable, creative, dreamy, energized, euphoric, focused, giggly, happy, light, motivated, pain-free, positive, refreshed, relaxed, talkative, upbeat, and less aware of pain), neutral (couch locked, foggy, forgetful, hungry, lethargic, red-eyes, restless, sleepy, thirsty, tired, and zoned out), and negative (anxious, dizzy, headache, nauseous, paranoid, and racing heart) emotives.

The proportion of individuals reporting an emotive effect was based on the percent of patients who reported that specific effect at least once.

### Data analysis

To test differences in mean anxiety response between males and females, independent sample t-tests were conducted. The core analysis examined within subject changes overtime (pre-medication to post-medication) and interactions overtime with two candidate moderators [gender (male, female), and age (18–29, 30–39, and 40 + years old)] by using analysis of variance (ANOVA). For significant main effects of interactions, post-hoc tests were conducted using a Bonferroni correction. A secondary analysis examined differences in proportion of emotives endorsed as a function of gender or age using chi-square test of independence. All analyses were conducted using IBM SPSS 28 statistics.

## Results

### Participants

Participants (*n* = 184) were a voluntary sample of Canadian medical cannabis consumers (61% female; 34.7 ± 8.0 years old) with 1028 tracked inhalation sessions using Aurora Cannabis dried flower. The average number of sessions per participant was 6, with a range of 1–117 sessions. An overview of the patient cohort can be found in Table 1.

**Table 1:** Overview of the patient cohort. The asterisk denotes *p* < 0.05 for the comparison between males and females. One participant logged 7 sessions and reported their gender as “unknown.” Their data was included in the total but not in either the male or female categories

Characteristic	Total (*n* = 184)	Males (*n* = 70)	Females (*n* = 113)
**Age (M ± SD)**	34.7 (± 8.0)	37.2 (± 9.1)	33.0 (± 6.7)
**Pre-medication anxiety score**	5.9 (± 2.2)	6.0 (± 2.1)	5.8 (± 2.3)
**Post-medication anxiety score**	2.2 (± 1.9)	2.1 (± 1.6)	2.3 (± 2.1)
**Average months anxiety users were using app**	3.6 (± 6.9)	3.4 (± 7.2)	3.6 (± 6.6)
**Average number of inhalations**	10.2 (± 6.8)	11.1 (± 7)*	9.7 (± 6.5)
**Average number of clinical indications per user**	5.4 (± 4.7)	4.4 (± 3.6)	6.1 (± 5.2)
**Average Efficacy (%)**	50.0 (± 0.23)	50.3 (± 0.23)	49.9 (± 0.23)

### The impact of medical cannabis as a function of gender

**Fig. 1:**
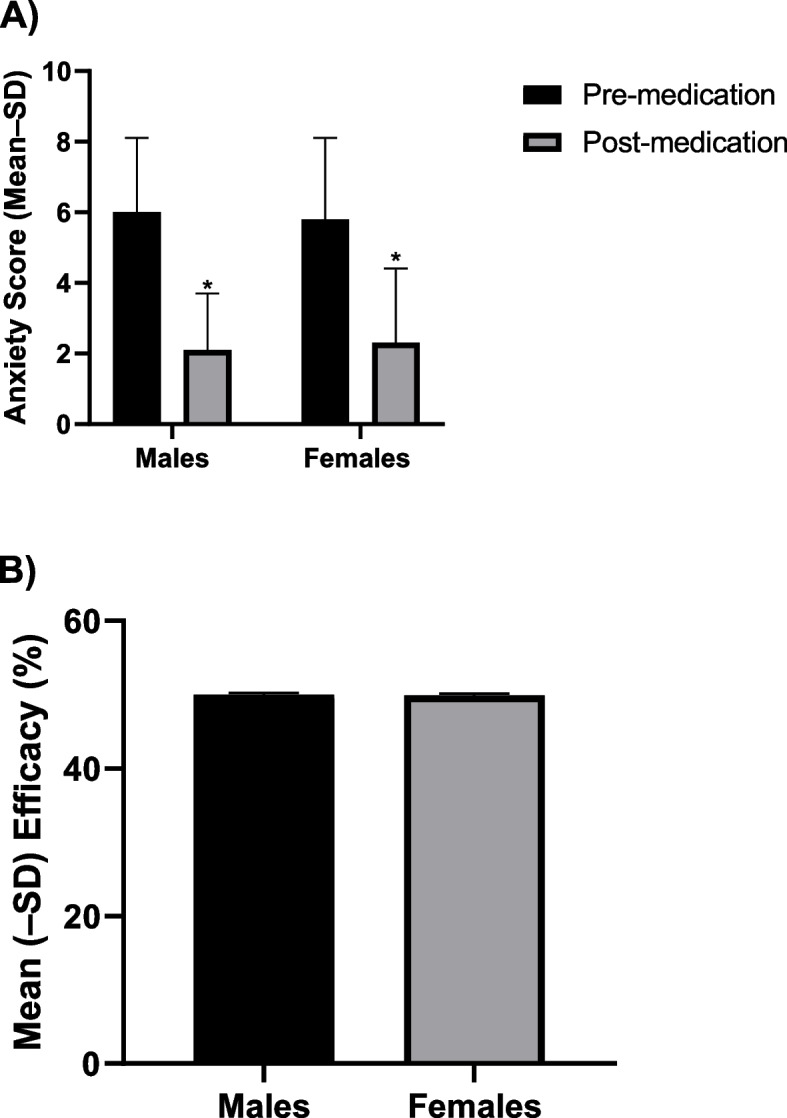
Mean (± SD) self-reported anxiety pre- and post-medication (**A**) and mean (± SD) percent efficacy (**B**) among males and females. The asterisk denotes *p* < 0.05 between pre- and post-medication (**A**)

Changes in anxiety (pre- to post-medication) and interactions with gender are shown in Table 2 and Fig. 1. An ANOVA revealed a significant main effect of Time [*F*(1, 1019) = 2987.12, *p* < 0.001] and significant Time × Gender interaction [*F*(1, 1019) = 4.02, *p *< 0.05]. Post hoc tests revealed a significant decrease in anxiety scores pre- to post- medication among both males and females and an overall average efficacy of 50%.

**Table 2:** Anxiolytic effects and emotives of cannabis consumption as a function of gender. The asterisk denotes *p* < 0.05 for the comparison between males and females. One participant logged 7 sessions and reported their gender as “unknown.” Their data was included in the total but not in either the male or female categories

		**Total (*n* = 184)**	**Males (*n* = 70)**	**Females (*n* = 113)**	***t* or *χ***^**2**^	***p*-value**
**Number of logged sessions**		1028	399	622		
**Average efficacy (M ± SD; %)**		50.03 (± 0.23)	50.29 (± 0.23)	49.86 (± 0.23)	*t* = -0.29	*p* = 0.77
**Optimal dose (number of inhalations)**		5–7, 11–13	9–11	5–7		
**Percent reporting positive emotives (%)**		94.8	100*	91.3	*χ*^2^ = 4.99	*p* = 0.03
	**Top 1 specific positive emotive (%)**	Relaxed (80)	Relaxed (83)	Relaxed (78)	*χ*^2^ = 0.68	*p* = 0.41
	**Top 2 specific positive emotive (%)**	Comfortable (57.5)	Comfortable (57.4)	Comfortable (57.5)	*χ*^2^ = 0.00	*p* = 0.99
	**Top 3 specific positive emotive (%)**	Happy (44.0)	Happy (50.0)	Happy (40.0)	*χ*^2^ = 1.31	*p* = 0.25
**Number of different positive side effects selected (out of 17)**		17	17	17		
**Average number reported positive emotives (M ± SD)**		4.54 ± 2.91	4.65 ± 3.08	4.45 ± 2.79	*t* = -0.37	*p* = 0.71
**Percent reporting negative emotives (%)**		12.7	11.1	13.8	*χ*^2^ = 0.20	*p* = 0.65
	**Top 1 specific negative emotive (%)**	Anxious (8.2)	Anxious (5.6)	Anxious (10.0)	*χ*^2^ = 0.85	*p* = 0.36
	**Top 2 specific negative emotive (%)**	Dizzy (4.5)	Dizzy (3.7)	Dizzy (5.0)	*χ*^2^ = 0.13	*p* = 0.72
	**Top 3 specific negative emotive (%)**	Headache (3.7)	Nauseous (3.7) Paranoid (3.7)	Headache (5.0) Paranoid (2.5) Nauseous (2.5)	Nauseous *χ*^2^ = 0.16 Paranoid *χ*^2^ = 0.16 Headache *χ*^2^ = 0.89	Nauseous *p* = 0.69 Paranoid *p* = 0.69 Headache *p* = 0.35
**Number of different negative side effects selected (out of 6)**		6	6	6		
**Average number of reported negative emotives (M ± SD)**		2.00 ± 1.32	1.83 ± 1.30	2.09 ± 1.38	*t *= 0.37	*p* = 0.71
**Percent reporting neutral emotives (%)**		63.4	59.3	66.3	*χ*^2^ = 0.68	*p* = 0.41
	**Top 1 specific neutral emotive (%)**	Thirsty (36.6)	Thirsty (33.3)	Thirsty (38.8)	*χ*^2^ = 0.41	*p* = 0.52
	**Top 2 specific neutral emotive (%)**	Sleepy (29.1)	Sleepy (20.4)	Sleepy (35.0)	*χ*^2^ = 3.34	*p* = 0.07
	**Top 3 specific neutral emotive (%)**	Hungry (24.6)	Hungry (20.4)	Hungry (27.5)	*χ*^2^ = 0.88	*p* = 0.35
**Number of different neutral side effects selected (out of 12)**		12	10	11		
**Average number of reported neutral emotives (M ± SD)**		3.49 ± 1.61	3.44 ± 1.54	3.53 ± 1.66	*t* = 0.26	*p* = 0.80

The optimal dose needed to experience anxiety-relieving effects was 9–11 inhalations for males and 5–7 inhalations for females.

Approximately, 94.8% reported a positive post-medication experience from using cannabis in the treatment of anxiety (Table 2). Males reported significantly more positive emotives than females. The top three positive emotives reported were relaxed, comfortable and happy. 12.7% reported a negative post-medication experience and the top three were anxious, dizzy, and headache. 63.4% reported neutral post-medication experiences and the top three were thirsty, sleepy, and hungry.

### THC indica cultivar

**Fig. 2:**
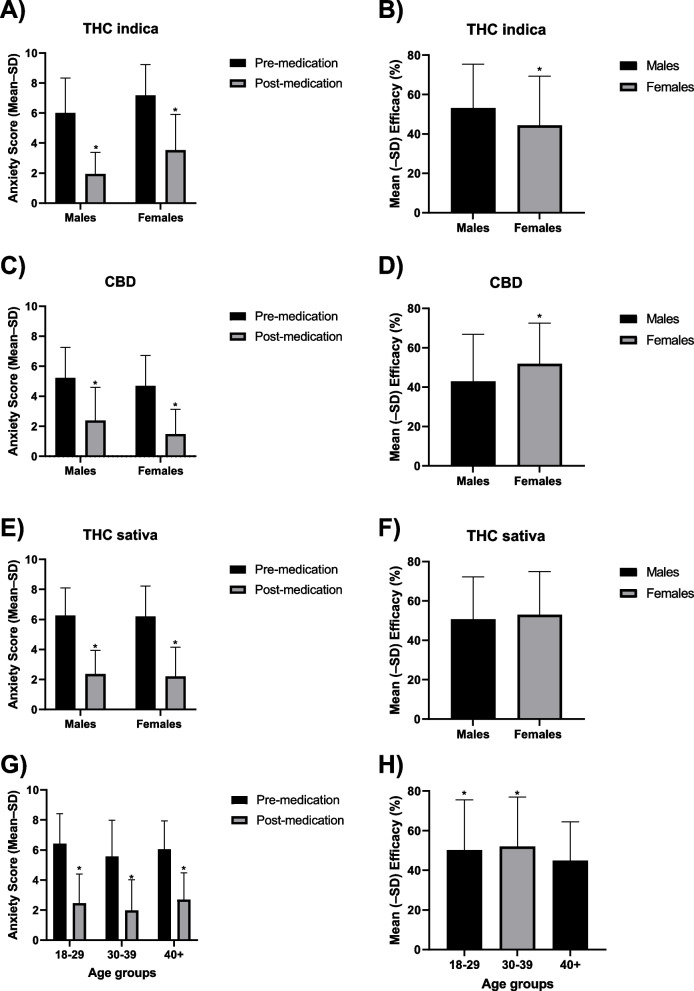
Mean (± SD) self-reported anxiety pre- and post-medication (**A**, **C**, and **E**) and mean (± SD) percent efficacy (**B**, **D**, and **F**) among males and females across three products THC indica (**A** and **B**), CBD (**C** and **D**), and THC sativa (**E** and **F**). Mean (± SD) self-reported anxiety pre- and post-medication (**G**) and mean (± SD) percent efficacy (**H**) among those aged 18–29, 30–39, and 40 + years old. The asterisk denotes *p* < 0.05 between pre- and post-medication (**A**, **C**, **E**, and **G**), between males and females (**B**, **F**), and between 18 and 29 years in comparison to 40 + years and 30–39 years in comparison to 40 + years (**H**)

Changes in anxiety (pre- to post-medication) and interactions with gender for the THC indica cultivar are shown in Table 3 and Fig. 2A and B. An ANOVA revealed a significant main effect of Time [*F*(1,340) = 963.19, *p* < 0.001] and Gender [*F*(1,340) = 52.34, *p* < 0.001]. An examination of marginal means revealed that there was a significant decrease in anxiety scores from pre- to post-medication and females were more likely to be anxious than males. Overall, the average efficacy was 48.3% and males reported a significantly higher efficacy than females.

**Table 3:** Anxiolytic effects and emotives of consuming a THC indica cultivar as a function of gender. The asterisk denotes *p *< 0.05 for the comparison between males and females. One participant logged 2 sessions and reported their gender as “unknown.” Their data was included in the total but not in either the male or female categories

		**Total (*n* = 93)**	**Male (*n* = 30)**	**Female (*n* = 62)**	***t* or *** χ*^**2**^	***p*-value**
**Number of logged sessions**		344	156	186		
**Pre-medication anxiety score**		6.65 (± 2.25)	6.01 (± 2.33)*	7.18 (± 2.04)	*t* = 4.88	*p* = 0.000002
**Post-medication anxiety score**		2.81 (± 2.15)	1.95 (± 1.43)*	3.53 (± 2.38)	*t* = 7.584	*p* < 0.000001
**Average efficacy (M ± SD; %)**		48.33 (± 24.17)	53.18 (± 22.21)*	44.27 (± 25.03)	*t* = -3.487	*p* = 0.0006
**Optimal dose (number of inhalations)**		10–11	10–11	10–11		
**Percent reporting positive emotives (%)**		91.0	100	86.1	*χ*^2^ = 3.68	*p* = 0.06
	**Top 1 specific positive emotive (%)**	Relaxed (79.1)	Relaxed (87.5)	Relaxed (74.4)	*χ*^2^ = 1.60	*p* = 0.21
	**Top 2 specific positive emotive (%)**	Comfortable (53.7)	Comfortable (50.0)	Comfortable (55.8)	*χ*^2^ = 0.21	*p* = 0.65
	**Top 3 specific positive emotive (%)**	Happy (35.8)	Happy (41.7) Light (41.7)	Happy (32.6) Light (23.3)	Happy * χ*^2^ = 0.56, Light * χ*^2^ = 2.49	Happy *p* = 0.46 Light *p* = 0.11
**Number of different positive side effects selected (out of 17)**		17	17	16		
**Average number reported positive emotives (M ± SD)**		3.67 ± 2.24	4.00 ± 2.60	3.46 ± 1.98	t = -0.87	*p* = 0.39
**Percent reporting negative emotives (%)**		7.5	4.2	9.3	*χ*^2^ = 0.588	*p* = 0.44
	**Top 1 specific negative emotive (%)**	Anxious (4.5)	Dizzy (4.2)	Anxious (7.0)		
	**Top 2 specific negative emotive (%)**	Dizzy (4.5)	NA	Dizzy (4.7)	Dizzy *χ*^2^ = 0.01	*p* = 0.93
	**Top 3 specific negative emotive (%)**	Headache (1.5)	NA	Headache (2.3)		
**Number of different negative side effects selected (out of 6)**		6	1	6		
**Average number of reported negative emotives (M ± SD)**		2.00 ± 1.40	Avg = 1	2.25 ± 1.41	NA	
**Percent reporting neutral emotives (%)**		71.6	70.8	72.1	*χ*^2^ = 0.003	*p* = 0.96
	**Top 1 specific negative emotive (%)**	Sleepy (38.8)	Thirsty (45.8)	Sleepy (41.8)	Sleepy *χ*^2^ = 0.47	Sleepy *p* = 0.49
	**Top 2 specific negative emotive (%)**	Thirsty (35.8)	Sleepy (33.3)	Thirsty (39.5)	Thirsty *χ*^2^ = 0.25	Thirsty *p* = 0.62
	**Top 3 specific negative emotive (%)**	Hungry (34.3)	Hungry (29.2)	Hungry (37.2)	Hungry *χ*^2^ = 0.21	Hungry *p* = 0.65
**Number of different neutral side effects selected (out of 12)**		10	9	10		
**Average number of reported neutral emotives (M ± SD)**		2.33 ± 1.48	2.53 ± 1.77	2.23 ± 1.31	*t* = -0.62	*p* = 0.54

The optimal dose needed to experience anxiety-relieving effects was 10–11 inhalations for both sexes.

91.0% reported positive post-medication experiences and the top three positive emotives reported were relaxed, comfortable and happy. 7.5% reported negative post-medication experiences and the top three negative emotives were anxious, dizzy, and headache. Males reported dizzy as the only negative emotive. 72.6% reported neutral post-medication experiences and the top three neutral emotives were thirsty, sleepy, and hungry.

### CBD cultivar

Changes in anxiety (pre- to post-medication) and interactions with gender for the CBD cultivar are shown in Table 4 and Fig. 2C and D. An ANOVA revealed a significant main effect of Time [*F*(1,367) = 617.90, *p* < 0.001]. An examination of marginal means revealed that there was a significant decrease in anxiety scores from pre- to post- medication. Overall, the average efficacy was 50.2% and females reported significantly higher efficacy than males.

**Table 4:** Anxiolytic effects and emotives of consuming a CBD cultivar as a function of gender. The asterisk denotes* p* < 0.05 for the comparison between males and females

		**Total (*n* = 69)**	**Male (*n* = 26)**	**Female (*n* = 43)**	***t* or ***χ*^**2**^	***p*-value**
**Number of logged sessions**		369	70	299		
**Pre-medication anxiety score**		4.79 (± 2.03)	5.23 (± 2.02)*	4.69 (± 2.03)	*t* = -2.002	*p* = 0.048
**Post-medication anxiety score**		1.66 (± 1.80)	2.39 (± 2.21)*	1.49 (± 1.65)	*t* = -3.197	*p* = 0.002
**Average efficacy (M ± SD; %)**		50.18 (± 21.53)	42.90 (± 23.86)*	51.88 (± 20.62)	*t* = 2.905	*p* = 0.005
**Optimal dose (number of inhalations)**		5–7	8–10	5–7		
**Percent reporting positive emotives (%)**		100	100	100		
	**Top 1 specific positive emotive (%)**	Relaxed (80.0)	Relaxed (83.3)	Relaxed (77.4)	*χ*^2^ = 0.30	*p* = 0.59
	**Top 2 specific positive emotive(%)**	Comfortable (58.2)	Comfortable (58.3)	Comfortable (58.6)	*χ*^2^ = 0.00	*p* = 0.98
	**Top 3 specific positive emotive(%)**	Light (45.5)	Happy (54.2) Light (45.8)	Light (35.5) Happy (35.5)	Happy *χ*^2^ = 1.92 Light *χ*^2^ = .002	Happy *p* = 0.17 Light *p* = 0.96
**Number of different positive side effects selected (out of 17)**		17	17	16		
**Average number reported positive emotives (M ± SD)**		4.11 ± 2.42	3.96 ± 2.51	4.23 ± 2.39	t = 0.4	*p* = 0.69
**Percent reporting negative emotives (%)**		12.7	12.5	12.9	*χ*^2^ = 0.002	*p* = 0.96
	**Top 1 specific negative emotive (%)**	Anxious (5.5)	Anxious (4.2%	Anxious (6.5)	*χ*^2^ = 0.14	*p* = 0.71
	**Top 2 specific negative emotive (%)**	Headache (5.5)	Headache (4.2)	Headache (6.5)	*χ*^2^ = 0.14	*p* = 0.71
	**Top 3 specific negative emotive (%)**	Dizzy (3.6)	Dizzy (4.2)	Dizzy (3.2)	*χ*^2^ = 0.03	*p* = 0.85
**Number of different negative side effects selected (out of 6)**		5	5	5		
**Average number of reported negative emotives (M ± SD)**		1.71 ± 1.25	1.67 ± 1.15	1.75 ± 1.50	*t* = 0.08	*p* = 0.94
**Percent reporting neutral emotives (%)**		45.5	41.2	48.2	*χ*^2^ = 0.25	*p* = 0.62
	**Top 1 specific negative emotive (%)**	Thirsty (32.7)	Thirsty (29.2)	Thirsty (35.5)	*χ*^2^ = 0.25	*p* = 0.62
	**Top 2 specific negative emotive (%)**	Hungry (16.4)	Hungry (16.7)	Sleepy (22.6)	Hungry *χ*^2^ = 0.003	Hungry *p* = 0.96
	**Top 3 specific negative emotive (%)**	Sleepy (16.4)	Sleepy (8.3)	Hungry (16.1)	Sleepy *χ*^2^ = 2.01	Sleepy *p* = 0.16
**Number of different neutral side effects selected (out of 12)**		11	6	11		
**Average number of reported neutral emotives (M ± SD)**		2.04 ± 1.53	1.5 ± 0.7	2.41 ± 1.80	t = 1.98	*p* = 0.06

The optimal dose needed to experience anxiety-relieving effects was 8–10 inhalations for males and 5–7 inhalations for females.

100% of males and females reported positive post-medication experiences and the top three positive emotives reported were relaxed, comfortable and light. 12.7% reported negative post-medication experience and the top three negative emotives reported were anxious, headache, and dizzy. 45.5% reported neutral emotives and the top three neutral emotives reported were thirsty, hungry, and sleepy.

### THC sativa cultivar

Changes in anxiety (pre- to post-medication) and interactions with gender for the THC sativa cultivar are shown in Table 5 and Fig. 2E, F. An ANOVA revealed a significant main effect of Time [*F*(1,308) = 11.71, *p* < 0.001]. An examination of marginal means revealed that there was a significant decrease in anxiety scores from pre- to post-medication. Overall, the average efficacy was 51.7%.

**Table 5:** Anxiolytic effects and emotives of consuming a THC sativa cultivar as a function of gender. The asterisk denotes *p* < 0.05 for the comparison between males and females. One participant logged 5 sessions and reported their gender as “unknown.” Their data was included in the total but not in either the male or female categories

		**Total (*n* = 70)**	**Male (*n* = 33)**	**Female (*n* = 36)**	***t* or ***χ*^**2**^	***p*-value**
**Number of logged sessions**		315	173	137		
**Pre-medication anxiety score**		6.24 (± 1.92)	6.27 (± 1.83)	6.20 (± 2.02)	*t* = -0.31	*p* = 0.76
**Post-medication anxiety score**		2.30 (± 1.75)	2.37 (± 1.57)	2.20 (± 1.95)	*t* = -0.808	*p* = 0.42
**Average efficacy (M ± SD; %)**		51.72 (± 21.68)	50.66 (± 21.53)	53.06 (± 21.86)	*t* = 0.964	*p* = 0.34
**Optimal dose (number of inhalations)**		4–7	4–5	6–7		
**Percent reporting positive emotives (%)**		97.9	100	96.0	*χ*^2^ = 0.94	*p* = 0.33
	**Top 1 specific positive emotive (%)**	Relaxed (66.7)	Relaxed (73.9)	Relaxed (60.0)	*χ*^2^ = 1.04	*p* = 0.31
	**Top 2 specific positive emotive (%)**	Comfortable (62.5)	Comfortable (69.6)	Comfortable (56.0)	*χ*^2^ = 0.94	*p* = 0.33
	**Top 3 specific positive emotive (%)**	Happy (52.1)	Happy (56.5)	Happy (48.0)	*χ*^2^ = 0.35	*p* = 0.55
**Number of different positive side effects selected (out of 17)**		17	17	16		
**Average number reported positive emotives (M ± SD)**		4.8 ± 2.9	5 ± 2.8	4.6 ± 2.9	*t* = -0.60	*p* = 0.55
**Percent reporting negative emotives (%)**		14.5	8.7	20	*χ*^2^ = 1.23	*p* = 0.27
	**Top 1 specific negative emotive (%)**	Anxious (10.4)	Anxious (8.7)	Anxious (12.0)	*χ*^2^ = 0.01	*p* = 0.91
	**Top 2 specific negative emotive (%)**	Racing heart (6.3)	Racing heart (4.3)	Racing heart (8.0)	*χ*^2^ = 0.27	*p* = 0.6
	**Top 3 specific negative emotive (%)**	Dizzy (2.1) Headache (2.1) Nauseous (2.1) Paranoid (2.1)	Nauseous (4.3 Paranoid (4.3) Dizzy (0) Headache (0)	Dizzy (4.0) Headache (4.0) Nauseous (0) Paranoid (0)	Dizzy *χ*^2^ = 0.94 Headache *χ*^2^ = 0.94 Nauseous *χ*^2^ = 1.11 Paranoid *χ*^2^ = 1.11	Dizzy *p* = 0.33 Headache *p* = 0.33 Nauseous *p* = 0.29 Paranoid *p* = 0.29
**Number of different negative side effects selected (out of 6)**		6	4	4		
**Average number of reported negative emotives (M ± SD)**		1.7 ± 1.1	2.5 ± 2.1	1.4 ± 0.5	*t* = -1.23	*p* = 0.27
**Percent reporting neutral emotives (%)**		58.3	60.9	56	*χ*^2^ = 0.12	*p* = 0.73
	**Top 1 specific negative emotive (%)**	Thirsty (35.4)	Thirsty (34.8)	Thirsty (36.0)	*χ*^2^ = 0.01	*p* = 0.93
	**Top 2 specific negative emotive (%)**	Hungry (25.0)	Hungry (26.1)	Hungry (24.0)	*χ*^2^ = 0.03	*p* = 0.87
	**Top 3 specific negative emotive (%)**	Restless (14.6) Sleep (14.6)	Restless (17.4) Sleepy (8.7)	Sleepy (20.0) Restless (12.0)	Restless *χ*^2^ = 0.28 Sleepy *χ*^2^ = 1.30	Restless *p* = 0.6 Sleepy *p* = 0.27
**Number of different neutral side effects selected (out of 12)**		11	11	10		
**Average number of reported neutral emotives (M ± SD)**		2.2 ± 1.3	2.2 ± 1.1	2.2 ± 1.6	*t* = -0.03	*p* = 0.98

The optimal dose needed to experience anxiety-relieving effects was 4–5 inhalations for males and 6–7 inhalations for females.

97.9% reported positive post-medication experiences and the top three positive emotives reported were relaxed, comfortable, and happy. 14.5% reported negative post-medication experiences and the top two negative emotives reported were anxious and racing heart. 58.3% reported neutral post-medication experiences and the top two neutral emotives reported were thirsty and hungry.

### The impact of age on efficacy of cannabis for anxiety

Changes in anxiety (pre- to post-medication) and interactions with age are shown in Table 6 and Fig. 2G, H. An ANOVA revealed a significant main effect of Time [*F*(1,1024) = 2519.91, *p* < 0.001] and Age [*F*(3,1024) = 15.24, *p *< 0.001], as well as a significant Time × Age interaction [*F*(3,1024) = 4.79, *p* < 0.01]. Post hoc tests revealed that there was a significant decrease in anxiety scores pre- to post-medication in all age groups. Those 30–39 years old had the lowest anxiety scores at both time points. The 40 + years old group (average age 46 years, ranging from 40 to 66 years) reported a significantly lower efficacy than the other age groups.

**Table 6:** Anxiolytic effects and emotives of cannabis consumption as a function of age. The asterisk denotes *p* < 0.05 between 30 and 39 years and both other age groups, ^ denotes *p* < 0.05 between 40 + and both other age groups and ^#^ denotes *p *< 0.05 between 18–29 years and both other age groups. Three participants who logged 23 sessions were not included in this table as they did not report their age. One female participant is included in both the 30–39 years and 40 + years as she logged sessions as a 39- and 40-year-old

		**18–29 years (*n* = 53)** **(M ± SD)**	**30–39 years (*n* = 88)** **(M ± SD)**	**40 + years (*n* = 41)** **(M ± SD)**	**ANOVA or ***χ*^**2**^	***p*-value**
**Gender**		33 females and 20 males	56 females and 32 males	25 females and 16 males		
**Number of logged sessions**		196	558	251		
**Pre-medication anxiety score**		6.43 (± 1.99)	5.58 (± 2.40) *	6.05 (± 1.90)	*F* = 12.10	*p* < 0.001
**Post-medication anxiety score**		2.46 (± 1.94)	1.98 (± 2.03) *	2.70 (± 1.79)	*F* = 13.28	*p* < 0.001
**Average efficacy (%)**		50.2 (± 25.4)	52.0 (± 25.0)	44.8 (± 19.6)^	*F* = 9.14	*p* < 0.001
**Optimal dose (number of inhalations)**		4–6	5–7, 11–13	4–5		
**Percent reporting positive emotives (%)**		85.7^#^	98.5%	96.6%	*χ*^2^ = 7.81	*p* = 0.02
	**Top 1 specific negative emotive (%)**	Relaxed (62.9%)	Relaxed (86.8%)	Relaxed (82.8%)	*χ*^2^ = 8.35	*p* = 0.02
	**Top 2 specific negative emotive (%)**	Comfortable (51.4)	Comfortable (57.4)	Comfortable (65.5)	*χ*^2^ = 1.29	*p* = 0.52
	**Top 3 specific negative emotive (%)**	Happy (40.0)	Happy (45.6) Light (39.7)	Happy (41.4) Light (48.3)	Happy *χ*^2^ = 0.34 Light *χ*^2^ = 3.63	Happy *p* = 0.84 Light *p* = 0.16
**Number of different positive side effects selected (out of 17)**		17	17	16		
**Average number reported positive emotives (M ± SD)**		4.3 ± 3.0	4.5 ± 2.9	4.4 ± 2.6	*F* = 0.09	*p* = 0.92
**Percent reporting negative emotives (%)**		22.9	7.4	10.34	*χ*^2^ = 5.33	*p* = 0.07
	**Top 1 specific negative emotive (%)**	Headache (11.4)	Anxious (5.9)	Anxious (10.3)	Anxious *χ*^2^ = 0.65 Headache *χ*^2^ = 7.75	Anxious *p* = 0.72 Headache *p* = 0.02
	**Top 2 specific negative emotive (%)**	Anxious (8.6%	Racing Heart (4.4)	Dizzy (3.5)	Dizzy *χ*^2^ = 1.79	Dizzy *p* = 0.41
	**Top 3 specific negative emotive (%)**	Dizzy (8.6) Nauseous (8.6) Racing Heart (2.9)	Dizzy (2.9) Headache (1.5) Nauseous (1.5)	Paranoid (3.5) Nauseous (0) Racing Heart (0)	Paranoid *χ*^2^ = 1.44 Nauseous* χ*^2^ = 5.13 Racing Heart *χ*^2^ = 1.35	Paranoid *p* = 0.49 Nauseous *p* = 0.08 Racing heart *p* = 0.51
**Number of different negative side effects selected (out of 6)**		6	6	3		
**Average number of reported negative emotives (M ± SD)**		2 ± 1.4	2.4 ± 1.5	1.6 ± 1.1	*F* = 0.27	*p* = 0.77
**Percent reporting neutral emotives (%)**		60.0	60.3	72.4	*χ*^2^ = 1.45	*p* = 0.48
	**Top 1 specific neutral emotive (%)**	Thirsty (42.9)	Thirsty (32.4)	Thirsty (37.9)	*χ*^2^ = 1.14	*p* = 0.57
	**Top 2 specific neutral emotive (%)**	Hungry (28.6)	Sleepy (26.5)	Sleepy (37.9)	Sleepy *χ*^2^ = 1.30	*p* = 0.52
	**Top 3 specific neutral emotive (%)**	Sleepy (28.6) Couchlocked (22.9)	Hungry (20.6) Couchlocked (14.7)	Hungry (24.1) Couchlocked (0)	Hungry *χ*^2^ = 0.83 Couchlocked *χ*^2^ = 7.17	Hungry *p* = 0.66 Couchlocked *p* = 0.03
**Number of different neutral side effects selected (out of 12)**		10	11	10		
**Average number of reported neutral emotives (M ± SD)**		3.4 ± 1.9**	2.3 ± 1.7	2.1 ± 1.5	*F* = 3.43	*p* = 0.04

The optimal dose needed to experience anxiety-relieving effects was 4–6 inhalations for 18–29-year-old, 5–7 and 11–13 inhalations for 30–39 years old, and 4–5 inhalations for 40 + years old.

There was a significant difference between age groups in the proportion of individuals reporting positive emotives. Those aged 18–29 years old were less likely to report a positive post-medication experience than those in the older age categories. Those aged 30–39 and 40 + years old were more likely to report relaxed as a positive emotive.

## Discussion

As approximately a third of patients diagnosed with anxiety will not respond to the standard treatment options (known as “treatment-refractory anxiety”) and only ~ 50% will fully recover (Garakani et al. 2020), this study examined patient-reported data from Strainprint^®^ on the three Aurora Cannabis dried flower products most commonly consumed during anxiety related sessions. The anxiolytic effects of these different cultivars and the impact of gender and/or age on outcomes and dosages was examined.

Overall, cannabis administration significantly decreased anxiety. We identified some gender and age differences in efficacy, optimal dosing and proportion of emotives reported across the three cultivars. However, both genders and all age categories experienced significant anxiolytic effects and tolerated cannabis well.

Overall, an average reported efficacy of ~ 50% was reported after consuming cannabis and there were no gender-related efficacy differences. This was surprising given that Cuttler et al. previously reported females had a greater reduction in anxiety following cannabis than males (Cuttler, Spradlin, and McLaughlin 2018).This difference could be the result of variations between the two patient cohorts that were not captured in the data collected such as type and severity of anxiety, any other comorbidities, race, ethnicity and other medication use. Our male cohort also reported significantly more positive emotives than females, but no gender differences were observed for neutral and negative emotives. As females have shown to be more susceptible to medical cannabis-related adverse events than males (Aviram et al. 2021) this was a surprising finding. Nonetheless, from this data, it is clear that cannabis is an effective anxiolytic and well tolerated by both genders.

The overall optimal dosing for females was less than males, indicating females may need less cannabis to achieve the same anxiolytic effect. This is supported by the larger literature as when individuals are able to self-titrate, women smoked less cannabis and subsequently had lower levels of THC in the blood, while experiencing the same acute effects as males (Matheson et al. 2020). It is possible these differences are a result of variation in muscle mass and fat distribution between the genders (Fattore and Fratta 2010a). However, females reported higher levels of cannabinoids in blood and oral fluid post-consumption of a THC brownie in comparison to males though some of the males in the cohort weighed less than some females (Spindle et al. 2020). Similarly, controlling for body weight and peak blood concentrations did not impact the significant sex differences in subjective effects reported in another study (Sholler et al. 2021). Thus, further investigation is required to fully elucidate why cannabinoid dosing differs between the genders as it appears it is not solely the result of differences in muscle mass and fat distribution.

The specific efficacy and optimal dosing of the three cultivars and how these items may be impacted by gender were examined. While no gender-related efficacy differences were identified for THC sativa, males experienced significantly higher efficacy with THC indica than females and females had significantly higher efficacy than males with CBD. While it was surprising to find gender differences in one high THC cultivar and not the other, females have been reported to prefer consuming CBD over THC (Moltke and Hindocha 2021). While we noted gender differences in two of the cultivars, the efficacy was similar across the three, indicating no THC:CBD ratio was significantly more beneficial than another. Furthermore, we identified some gender differences in optimal dosing with males requiring less inhalations than females to achieve efficacy with THC sativa but requiring more inhalations than females for CBD.

The wider scientific literature supports our finding that all three cultivars provided significant anxiolytic effects as there is evidence for both CBD (Shannon et al. 2019; Rapin et al. 2021; Crippa et al. 2011; Bergamaschi et al. 2011) and THC (Fabre and McLendon 1981; Kamal et al. 2018; Skrabek et al. 2008; Cahill et al. 2021) to reduce anxiety. CBD has been shown to be an agonist at both cannabinoid receptors (CB; CB_1_ and CB_2_) and the serotonin-1 A (5HT_1A_) (Breuer et al. 2016; de Gregorio et al. 2019; Resstel et al. 2009) receptor while THC’s partial agonism at CB_1_ has been well documented (Petitet et al. 1998; Pertwee 2008). Given that both cannabinoids interact with CB_1_ receptors, these gender differences in efficacy and optimal dosing may be tied to gender-dependent differences in CB_1_ receptor expression and binding site density throughout the brain (Fattore and Fratta 2010b; Rubino et al. 2011). For instance, under healthy conditions, estradiol has been shown to increase CB_1_ receptor binding site density in the amygdala but reduced it in the hypothalamus (Riebe et al. 2010) and females have lower CB_1_ receptor binding density than males in numerous brain regions tied to anxiety (van Laere et al. 2008). Additionally, in response to chronic stress, CB_1_ receptors were downregulated in the hippocampus of male rodents but upregulated in females (Reich, Taylor, and McCarthy 2009).There are also documented gender differences in terms of serotonin signaling and 5HT_1A_ receptor expression (Toufexis et al. 2014; Goel, Innala, and Viau 2014). 5HT_1A_ receptor expression is regulated by estrogen and this interaction has been hypothesized to influence why females experience higher rates of mood disorders (Toufexis et al. 2014). Thus, these gender-based signaling differences provide some insight as to why the genders experienced differences in efficacy with the THC indica and CBD. However, why no gender differences were observed with the THC sativa cultivar is unclear. Therefore, further investigation is required to understand the mechanistic differences in the anxiolytic effects of cannabinoids between the genders.

We also found all age groups experienced significant anxiolytic effects with cannabis. The 30–39-year-old group reported a higher number of optimal inhalations than other groups, which may be the cause of the higher average efficacy and positive emotives observed in this group. Those 40 + reported a significantly lower efficacy for cannabis compared to those under 40. However, those 40 + were more likely to report positive emotives than 18–29-year-olds. Because polypharmacy is more prevalent in older populations (Rotermann et al. 2014) it is possible that this population experienced some drug-cannabis interactions that contributed to altered efficacy and side effects (Anderson and Chan 2016; Rong et al. 2018; Cital et al. 2021; Geffrey et al. 2015). While the 40 + group’s average age was only 46, data from Statistics Canada show consistent trends that as adults age, they are more likely to be prescribed medications than their younger counterparts; of those 18–39 years, 38% were prescribed medications versus 56% of those aged 40–59 years (Statistics Canada 2021). Furthermore, a number of studies have shown that older populations respond less effectively than younger populations to anxiety treatments (Wetherell et al. 2013; Carl et al. 2020) as well as brain activation patterns differ between older and younger populations in response to threat-related cues (Gold et al. 2020) that could explain the age differences we identified.

While data captured via the Strainprint^®^ app has been included in numerous peer-reviewed publications and the ability for patients to capture their experiences in real time is a strength of this method, especially given the cyclic nature of anxiety, this study is not without limitations. Firstly, using the Strainprint^®^ app rather than the use of validated questionnaires typically used in observational studies is a limitation of this study. Furthermore, participants self-reported their anxiety and the diagnosis was not confirmed by a healthcare practitioner. Additionally, due to the nature of this naturalistic study, we are unable to confirm if any pharmaceuticals and/or other cannabis products were being consumed in addition to the products participants logged sessions on, the average use of cannabis per month, the historical use of cannabis, the race and ethnicity of participants nor any diagnosed comorbidities. The ability to access medical cannabis could also have been a barrier due to an inability to find a physician to authorize medical cannabis. While Aurora Cannabis does offer a compassionate program for lower income patients and there is the option to claim medical cannabis as a medical expense on federal income taxes, widespread insurance coverage for medical cannabis is still lacking in Canada. Thus, without widespread insurance coverage, the costs of cannabis will continue to be a barrier for many to access this medicine. We also only focused on inhaled dried flower sessions, excluding other formats, which limited our sample size. As it has been reported females prefer ingestible formats (Cuttler, Mischley, and Sexton 2016), it is possible further gender differences may have been identified if we had included ingestible products. There have also been reports that bioavailability of inhaled cannabis significant differs between those who are heavy and light consumers; bioavailability for heavy consumers was reported as 23 ± 16% (Lindgren et al. 1981) and 27 ± 10% (Ohlsson et al. 1986) versus bioavailability for light consumers at 10 ± 7% (Lindgren et al. 1981) and 14 ± 1% (Ohlsson et al. 1986). Other studies have shown significant differences in THC and 11-OH-THC C_max_ between males and females post inhalation of cannabis (Chiang et al. 1982; Sholler et al. 2021). Thus, while dosing was reported as number of inhalations, it is possible the concentration of cannabinoids delivered differed between participants based upon comfortability with smoking or vaping and/or due to their gender, even if the number of inhalations were similar.

Overall, cannabis was effective in relieving anxiety and well-tolerated at the doses consumed, independent of CBD and THC ratios. While one cultivar was not significantly more effective than the others, we did identify some gender and age differences in optimal dosing across the three cultivars. We suggest that the outlined THC:CBD ratios and optimal inhalations may be used as a starting point for patients and healthcare practitioners looking to use cannabis as an anxiolytic in order to mitigate the trial-and-error aspect of initiating medical cannabis treatments. Additionally, we recommend the above dataset be used as the foundation for future clinical trials to fully elucidate the efficacy of cannabis for the management of anxiety under more controlled conditions.

## Data Availability

All data generated or analyzed during this study are included in this published article.

## Abbreviations

11-OH-THC: 11-Hydroxy-tetrahydrocannabinol
CBD: Cannabidiol
CB: Cannabinoid receptor
C_max_: Peak plasma concentration
5HT_1A_: Serotonin-1 A
THC: Tetrahydrocannabinol
T_max_: Time to peak plasma
